# The Effect of Emotional Content on Brain Activation and the Late Positive Potential in a Word n-back Task

**DOI:** 10.1371/journal.pone.0075598

**Published:** 2013-09-26

**Authors:** Juliane Kopf, Thomas Dresler, Philipp Reicherts, Martin J. Herrmann, Andreas Reif

**Affiliations:** 1 Department of Psychiatry, Psychosomatics and Psychotherapy, University of Würzburg, Würzburg Germany; 2 Department of Psychiatry and Psychotherapy, University of Tübingen, Tübingen, Germany; 3 Department of Psychology, Biological Psychology, University of Würzburg, Würzburg, Germany; Charité University Medicine Berlin, Germany

## Abstract

**Introduction:**

There is mounting evidence for the influence of emotional content on working memory performance. This is particularly important in light of the emotion processing that needs to take place when emotional content interferes with executive functions. In this study, we used emotional words of different valence but with similar arousal levels in an n-back task.

**Methods:**

We examined the effects on activation in the prefrontal cortex by means of functional near-infrared spectroscopy (fNIRS) and on the late positive potential (LPP). FNIRS and LPP data were examined in 30 healthy subjects.

**Results:**

Behavioral results show an influence of valence on the error rate depending on the difficulty of the task: more errors were made when the valence was negative and the task difficult. Brain activation was dependent both on the difficulty of the task and on the valence: negative valence of a word diminished the increase in activation, whereas positive valence did not influence the increase in activation, while difficulty levels increased. The LPP also differentiated between the different valences, and in addition was influenced by the task difficulty, the more difficult the task, the less differentiation could be observed.

**Conclusions:**

Summarized, this study shows the influence of valence on a verbal working memory task. When a word contained a negative valence, the emotional content seemed to take precedence in contrast to words containing a positive valence. Working memory and emotion processing sites seemed to overlap and compete for resources even when words are carriers of the emotional content.

## Introduction

Emotions are a ubiquitous component of everyday life, as they influence behavior to a large extent. Emotions act upon reasoning, memory, problem-solving and decision making [1]. The emotional content is processed with priority compared to other stimulus attributes such as shape or color [2,3]; however in many situations it is important to control emotion processing, e.g. when it interferes with task demands such as the storage, maintenance, and manipulation of information in working memory.

Working memory can be described as a system of limited capacity for temporary storage and manipulation of information [4], or as a process which holds information on-line, in order to guide behavior when no other external cues are present [5]. Working memory can be experimentally tested in multiple ways. In neuroimaging studies the n-back task is frequently used. Participants are confronted with a sequence of stimuli and are required to indicate if a stimulus is identical with the preceding one (1-back), with the one preceding the current by two (2-back), etc. Stimuli can be either verbal or nonverbal, and either the identity or the location of a stimulus can be monitored [6]. Working memory research involving emotions usually relies on the induction of emotion before the actual task [7]. Kensinger and Corkin [8] state two problems with this approach: mood induction might lead to the neglect of other stimulus dimensions and to the neglect of the task itself. Therefore, they [8] used a 2-back working memory task with emotional stimuli: first, emotional faces and then, emotional words, to circumvent those problems, and found that only emotional faces influence working memory performance. When the emotional information is presented in form of a word, however, the emotional content of the word plays no role in execution of the working memory task. It is also noteworthy that the paradigm of Kensinger and Corkin [8] categorized words not into positive, negative and neutral words but rather into arousing and neutral words. This is a crucial point when conducting a study with emotional words. Dresler et al. [9], for example, used an emotional Stroop task to show that arousal produces emotional interference independent of valence. Other studies however have demonstrated that the impact of arousal is different for negative and positive words [10], underscoring the importance to differentiate these conditions [11].

More recently, Levens and Phelps [12] reported that emotional content in a word can indeed influence working memory performance. Emotional compared to neutral words resulted in less interference in working memory tasks; interference conflicts were resolved faster for emotional words. However, Levens and Phelps only examined a processing component of the working memory task, the interference resolution. In a follow-up study, they demonstrated that the influence of emotional content on working memory can also be seen in activation patterns in the brain [13], and found greater activation patterns for emotional versus neutral words in an interference task. However, these differences were only seen in subcortical areas such as the amygdala. In the prefrontal cortex, the main area necessary for working memory function [6], the only difference in activation could be shown for the different interference levels. Levens and Phelps [13] concluded that the emotional component of working memory processes are processed in a network which includes the anterior insula, the amygdala and the right orbito-frontal cortex (OFC). Interestingly, Levens and Phelps did not distinguish different valences, only different arousal levels, and therefore categorized the words used in the task as either neutral or emotional. However, there is evidence from studies using electroencephalography (EEG) recordings showing that the valence of a word does make a difference in the processing of emotional stimuli in a working memory task: MacNamara and colleagues [14] performed an n-back task using emotional pictures as distractor material and found a modulation of the late positive potential (LPP) not only due to the emotional content of the picture, but also to the load of the working memory. The LPP was larger on low load trials compared to high load trials as well as on aversive trials compared to neutral trials.

The LPP is an event related potential (ERP) which constitutes a sustained positivity with a relatively late onset (circa 300 ms after the stimulus onset) and is proposed to be able to distinguish between the processing of emotional versus non-emotional stimuli. Compared to the non-emotional stimuli, the emotional stimuli have been shown to elicit a higher positivity in the EEG. The LPP has two components, an early and a late LPP, with the late LPP starting approximately 1000 ms after stimulus onset [15]. The LPP has been mainly observed in paradigms that use emotional pictures to test emotion regulation [15], but also in analyses of word processing [16].

There are many studies using emotional words to test for differences in the LPP [17,18]. However, no verbal working memory study has used the LPP to test for differences in the processing of emotional content. In our study, the LPP is used as a dissociative tool, adding to the information gathered by measuring the dorsolateral prefrontal cortex (dlPFC) activity.

After Considering All the Work Done so Far, We Propose the Following Study:

In order to investigate the effect of valence on working memory, the study uses words from the Berlin Affective Word List [11], which are classified not only for arousal and valence but have also been matched for properties like word length, syllable length and frequency. This extensive characterisation of the words made it possible to choose positive and negative words which do not differ in arousal but only in valence, and in consequence, all differences between words can be attributed to the difference in valence. Neutral words could, of course not be matched in arousal to the emotional words. This fact has to be considered when interpreting the results. All words were implemented in an n-back task containing an easy difficulty one-back condition, a medium difficulty two-back condition and a hard three-back condition. While conducting the test, near-infrared-spectroscopy (NIRS) was applied to measure changes in brain oxygenation levels. Even though all studies referenced in the introduction are fMRI studies, we are rather confident that the findings from these studies can be translated to fNIRS results. Both fMRI and fNIRS methods are based on the same underlying indirect measurement of neural activity (i.e. the BOLD effect), and there is growing evidence that results from fMRI studies can be translated to fNIRS [19]. The region of interest is the dlPFC. A simultaneous LPP is measured to investigate the possibility for generalisation of earlier findings concerning the LPP and emotional words.

In summary, our hypotheses are that not just arousal, but valence as well influences the outcome of a working memory test, as can be seen by differential brain activation in the dlPFC measured by functional NIRS, and that working memory tasks in turn influence the processing of emotion as measured by the LPP.

## Materials and Methods

### 1. Participants

The study was reviewed and approved by the Ethics Committee of the University of Wuerzburg, and all procedures were in accordance with the latest version of the Declaration of Helsinki. All test subjects gave written informed consent after comprehensive explanation of the experimental procedures.

Thirty-two healthy right-handed volunteers participated in the study. Two participants had to be excluded for technical reasons. The final sample thus consisted of 15 women and 15 men; mean age of the subjects was 23.6±2.7 (±standard deviation) years. To ensure comparable intelligence levels within the test population, subtest 3 of the Leistungsprüfsystem (LPS) [20], which measures nonverbal fluid intelligence, and the Mehrfachwahl-Wortschatz-Test (MWT-B) [21], which measures crystallized intelligence, were administered. This test can also attest to the vocabulary similarities among test subjects, all of which were native German speakers. For the LPS, the mean score was 32.2±3.8 and for the MWT-B the mean score was 30.2±3.3, indicating that all participants were of the same intelligence level. All participants were free of past and current axis I (classified via the DSM V) disorders as assessed with the MINI interview [22]. To control for their current emotional status, the German version of the Positive and Negative Affect Schedule (PANAS) [23] was filled out by the participants. The mean score for the positive scale of the PANAS was 29.2±4.5, and 10.7±2.1 for the negative effect scale (scores from 10 to 40 could be obtained), indicating that all participants shared equal starting conditions.

### 2. Emotional n-back task

Participants saw blocks of ten consecutive words presented on a computer screen. Two of these words were used as cues and were followed by a target word which was presented one, two or three words after the cue word, to vary the difficulty level and thus the working memory load. When the participants saw the target word, they were instructed to press the space key on a standard keyboard. Each block of ten words comprised one difficulty level. Also the emotional content of the words was varied; the words were positive, negative or neutral. Each block encompassed only one emotional valence. In total, there were three blocks where the emotional valence was negative and the difficulty level was one, meaning the target word followed the cue word directly. Three blocks had a negative emotional valence and the difficulty level was two, meaning there was one word between cue word and target word. Three blocks had a negative emotional valence and the difficulty level was three, meaning there were two words between the cue word and the target word. The same was true for the positive and the neutral emotional valence; therefore, in sum the experiment had 27 blocks. Each block was 20 seconds long, and blocks were separated by a 20-second pause. Within each block, each word was visible for 500 ms, followed by a blank screen, which was also 500 ms long. The difficulty level of the blocks was pseudorandomized, and difficulty levels always varied from one block to another. In the pause preceding a block, the instruction for the task (1-, 2-, 3-back) was presented on the screen.

The words (a list of which can be seen in Table 1) were chosen from the Berlin Affective Word List [11], were all nouns and matched for valence (positive nouns had a mean valence of 2, negative nouns a mean valence of -2 and neutral words a mean valence rating of 0, with the valence scale spanning from -3 for very negative to 3 for very positive). Mean arousal of emotional words was matched in respect of their valence, meaning the negative and positive words had a mean arousal of 3, the neutral words had a mean arousal of 0 (on a scale from 1: very low arousal to 5: very high arousal). To ensure equal working memory load for all words, phoneme length (5), syllable length (2), and number of letters (6) were matched as well.

**Table 1:** List of words used in the task.

**negative words**	**positive words**	**neutral words**
TYPHUS (typhus)	RETTER (savior)	ABLAUF (sequence)
BEFEHL (order)	WISSEN (knowledge)	AFFEKT (affect)
ARREST (warrant)	MUTTER (mother)	BANNER (banner)
TYRANN (tyrant)	GEWINN (win)	GERUCH (smell)
GREUEL (horror)	GEFÜHL (feeling)	KELLER (basement)
SEENOT (distress at sea)	HIMMEL (heaven)	LOSUNG (watchword)
HORROR (horror)	FRIEDE (peace)	STELLE (position)
TRAUMA (trauma)	URLAUB (vacation)	ZEUGIN (eye witness)
TERROR (terror)	SOMMER (summer)	AKZENT (accent)
UNHEIL (calamity)	FREUND (friend)	INHALT (content)

Before the experiment, participants practised the task using one block of each difficulty (1-, 2-, 3-back). Before the task itself, completed a training phase, where they saw 30 consecutive neutral words in the 1-back condition, the same neutral words in the 2-back condition and in the 3-back condition. After the experiment, participants were prompted to evaluate the words used for their arousal and valence (words could be rated from 1: very unpleasant to 9: very pleasant and 1: not arousing to 9: very arousing).

### 3. Functional Near-Infrared Spectroscopy

The basics of fNIRS are described in detail elsewhere [24,25] NIRS allows a highly reliable measurement of cortical activation [26,27]. We used an ETG-4000 Optical Topography system (Hitachi Medical Co., Japan) with a 52-channel array of optodes covering an area of 30 x 6cm of the forehead. The interoptode distance is 3 cm, in order to allow sufficient penetration depth. The array comprises of 17 light emitters (semiconductor laser) and 16 photo-detectors (Avalanche photodiodes). The photo-detectors collect the reflected near-infrared light of its surrounding emitters. A channel is defined as the measuring point of activation, which is the region between one emitter and one detector. The array was fastened to the head by elastic straps. The probe set was placed on the head so that detector optode 26 was on the position for Fpz and aligned to T3/T4 (for emitter optodes 28 and 23), according to the international 10-20 system for EEG electrode placement [28,29]. The array therefore covers both left and right frontal cortex areas.

### 4. Electroencephalogram

Continuous EEG was recorded from 5 scalp electrodes placed according to the 10/20 system [28,29]: Cz, CPz, Pz, CP1, CP2, and both mastoids. The ground electrode was placed on the left part of the scalp in the centroparietal region (corresponding to CP3). The electrooculogram (EOG) was recorded from four facial electrodes: vertical eye movements were measured with two electrodes placed approximately 1 cm above and below the right eye. Horizontal eye movements were measured using two electrodes placed 1 cm away from the outer canthi of each eye. ERPs were recorded with a 64-channel Quick Amp amplifier (Brain Products, Munich, Germany) and Vision Recorder software (version 2.0, Brain Products, Munich, Germany). Data were referenced online to an average reference including all electrodes. This average referencing is a built-in feature of the Quick Amp and cannot be changed. However, due to the small number of electrodes, the data were rereferenced offline, for details please see the data analysis section. Sampling rate was set to 1000 Hz. All channels were amplified with a band-pass from DC to 200 Hz. The impedances were kept below 5 kΩ.

### 5. Data Analysis

The program SPSS version 20 (IBM Corp. Released 2011. IBM SPSS Statistics for Windows, Version 20.0. Armonk, NY: IBM Corp.) was used for all statistical analyses. Details on the statistical tests used can be found in each subsection.

#### 5.1 Behavioral Data

3x3 repeated measures analyses of variance (ANOVA) for the difficulty-levels (1-back, 2-back and 3-back) by emotional valence (negative, positive and neutral) were conducted for the reaction time and the errors made in the experiment. In case of non-sphericity, Greenhouse-Geisser adjustment was used. This is true for all data analyses.

Functional Near-Infrared Spectroscopy- first, the high frequency portion of the signal was removed by applying a moving average (MA) filter with a time window of 1 s. To remove slow drifts, a 3 element discrete cosine transform basis set was then used on the data.

The last five seconds before a block was taken as baseline period. Thereafter, the last 15 seconds of the 20-second block were defined as the activation period, since 5 seconds after the initial trigger the neural response should already be seen in an increase in activation [30]. The mean of oxygenated haemoglobin [O_2_Hb] and deoxygenated haemoglobin [HHb] concentrations were computed for each segment and baseline-corrected.

**Figure 1:**
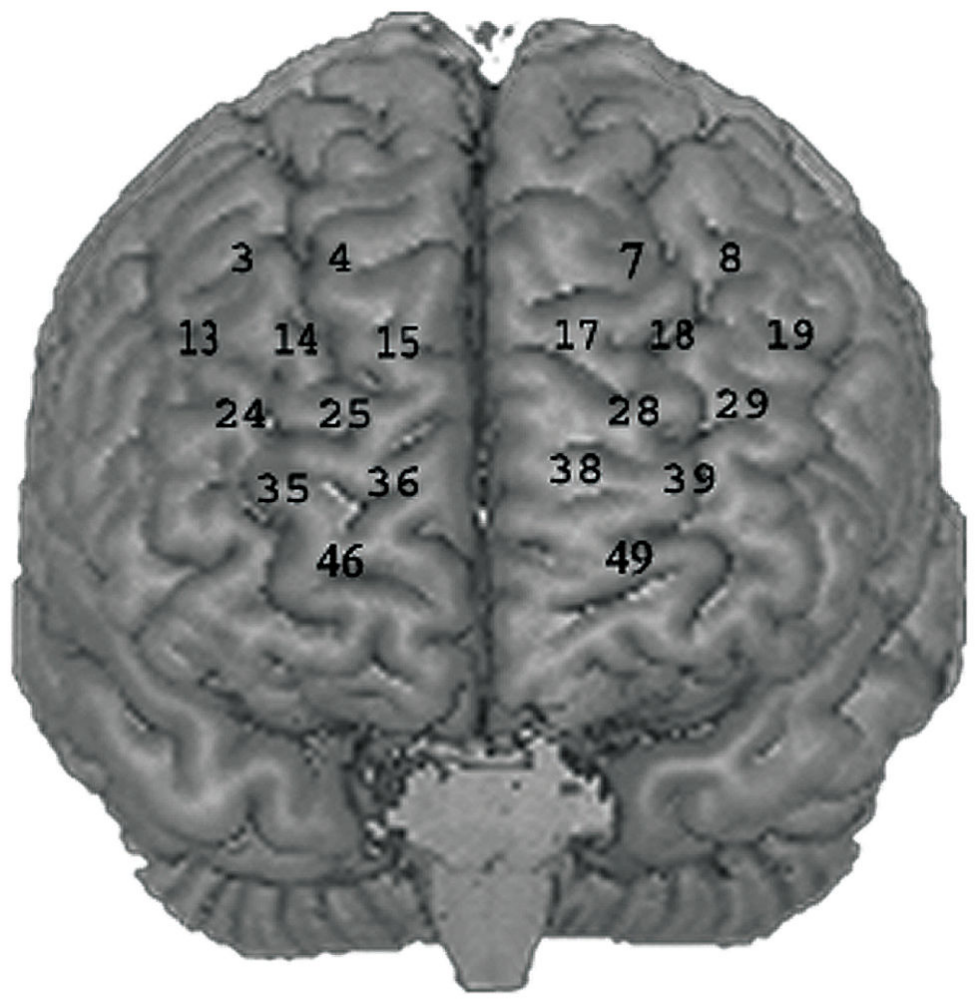
Optode Placement. Placement of the fNIRS channels included in the ROI analyses of oxygenated and deoxygenated haemoglobin concentration changes.

We assigned fNIRS channels to specific brain areas according to probabilistic maps [31] and defined regions of interest (ROIs) for both hemispheres. Based on previous work [6,32,33], we defined the dlPFC (Brodman areas 9/10/46) as ROIs. This corresponds to channels 3, 4, 13, 14, 15, 24, 25, 35, 36, 46 for the right dlPFC, and channels 7, 8, 17, 18, 19, 28, 29, 38, 39, 49 for the left dlPFC. For statistical analyses of the fNIRS data, 3x3 ANOVAs for each ROI were calculated; the within-factors were load (i.e. 1-back, 2-back and 3-back) and emotional valence (i.e. negative, neutral and positive). Subsequently, relevant post hoc tests were conducted (for details on channel placement and ROI location, see Figure 1).

#### 5.2 EEG

To ensure compatibility of our data with the already existing data from McNamara et al. [14] we used their analysis specifications. The data were analyzed using Vision Analyzer 2 software (Brain Vision, Munich). Offline, the data were filtered with a Butterworth Zero Phase Filter with a low cut-off of 0.1 Hz and a high cut-off of 20 Hz, eye blink and ocular corrections were conducted according to the Gratton, Coles and Donchin [34] algorithm.

The data were then segmented into time windows starting 100 ms before the reference marker (indicated by the onset of a word) and ending 1900 ms after the reference marker.

An automatic procedure built in the analyzer software detected and rejected artefacts: it was specified to reject any voltage steps more than 50.0 µV between sample points, a voltage difference of 300.0 µV within a trial, and a maximum voltage difference of less than 0.50 µV within 100 ms intervals.

After that, the data were re-referenced offline to a combined mastoid reference. Afterwards the baseline was corrected. The baseline, defined as 100 ms before word onset, was subtracted from the signal.

Because the LPP is maximal at centro-parietal sites [35-38], it was scored as the average activity from five centro-parietal sites (Pz, CPz, Cz, CP1 and CP2). Based on this research and following our presentation time of the stimuli of 500 ms, we evaluated the window of 350-600 ms after stimulus onset (mean activity). The late portion of the LPP was not analyzed because of the short time each word was visible. The target trials were too small in number and not analysed. In all, 24 trials of every condition went into the analysis.

For the statistical analysis, the same ANOVA as for the fNIRS data was calculated. Relevant post hoc tests were conducted.

## Results

### 1. Valence and Arousal Ratings

Repeated measures t-tests for the valence ratings showed significant differences for the valence between negative and neutral words (t(29) = 9, *p* < .001), between positive and neutral words (t(29) = 18.1, *p* < .001), and between positive and negative words

(t(29) = 18.3 *p* < .001), see also Table 2. Repeated measures t-tests for the arousal ratings showed significant differences for the arousal ratings between negative and neutral words (t(29) = 7.3, *p* < .001) and between the positive and neutral words (t(29) = 7.1, *p* < .001), but no significant differences between the positive and negative words (t(29) = 1.9, *p* > .05). The ratings completed by the participants were done in order to repeat the results from the original study by Vö et al [11,39], and to ensure that the actual arousal and valence ratings were equal to the ones already published.

**Table 2:** Means and standard deviations from the valence and arousal ratings of the words used in the paradigm.

**Words**	**Mean**	**SD**
**Arousal**		
Negative	5.24	1.49
Positive	4.68	1.47
Neutral	2.77	1.32
**Valence**		
Negative	2.58	0.79
Positive	7.62	0.55
Neutral	5.00	0.34

The means presented here are based on the ratings from our own sample with n=30. The means and standard deviations on which the words were selected were from the Berlin Affective Word List and can be found in the methods and materials section.

### 2. Behavioral Results

A 3x3 repeated measures ANOVA revealed a significant main effect of the difficulty level on the reaction time (F (1.4, 58) = 31.3, *p* < .001). A main effect for the factor valence (F(2, 58) < 1) or interaction effect could not be found (F(4, 116) < 1). Post hoc t-tests for the main effect difficulty revealed significant differences, indicating increasing reaction times with increasing difficulty. All tests were significant (t(29) > 4.4, all *p* < .001).

**Figure 2:**
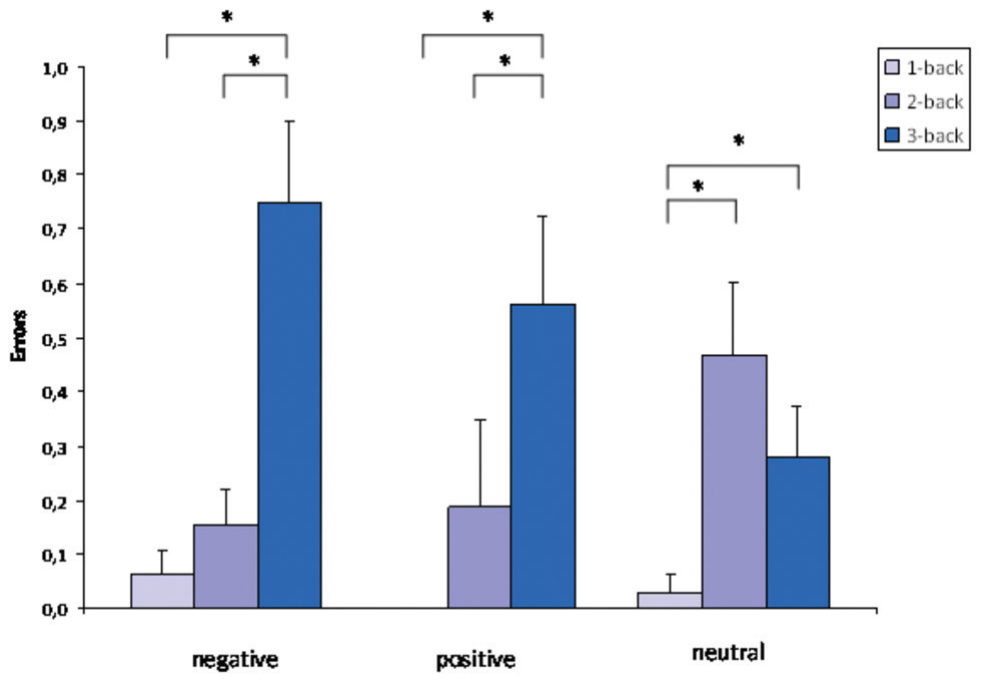
Number of errors. Errors made in the emotional n-back test in the different valence categories. Error bars indicate the standard error. An asterisk indicates a significant result.

The tests for normal distribution in the error data showed significant p-values for all differences (all *p* < .001). Non-parametric Wilcoxon tests however, show the same results as parametric t-tests for paired samples (see also Table 3). Because we were interested in interaction terms, and the ANOVA is robust against violations of assumptions for parametric testing for N > 20 we therefore calculated a 3x3 ANOVA. Results from that ANOVA should be interpreted carefully. The 3x3 ANOVA for the errors revealed a significant main effect for the difficulty level (F (1.5, 43.9) = 15.6, *p* < .001). No significant main effect for the valence could be found (F < 1). A significant interaction effect for the errors (F (2.8, 82.1) = 3.7, *p* = .016) was observed, indicating that word valence influenced the error rate differentially in each difficulty level, the more difficult the task; the more errors were made especially in the negative valence condition (see Figure 2). The details of the post hoc t-tests concerning the changes within the difficulty level are presented in Table 3.

**Table 3:** non-parametric Wilcoxon and parametric t-tests for errors.

**Errors**	**t-tests**					**Wilcoxon**	
**Negative**	**Mean**	**SD**	**T**	**df**	**Sig. (2-tailed)**	**Z**	**Sig.**
1-back vs. 2-back	-.09375	.46555	-1.139	29	.263	-1.342	.180
1-back vs. 3-back	-.68750	.89578	-4.342	29	**.000**	-3.397	**.001**
2-back vs. 3-back	-.59375	.87471	-3.840	29	**.001**	-3.132	**.002**
**Positive**							
1-back vs. 2-back	-.18750	.73780	-1.438	29	.161	-1.633	.102
1-back vs. 3-back	-.56250	.91361	-3.483	29	**.002**	-2.85	**.004**
2-back vs. 3-back	-.37500	.97551	-2.175	29	**.037**	-1.877	.060
**Neutral**							
1-back vs. 2-back	-.43750	.80071	-3.091	29	**.004**	-2.484	**.013**
1-back vs. 3-back	-.25000	.56796	-2.490	29	**.018**	-2.309	**.021**
2-back vs. 3-back	.18750	.89578	1.184	29	.245	-0.54	.589
**1-back**							
Negative vs. Positive	-.06250	.24593	-1.438	29	.161	-1	.317
Negative vs. Neutral	-.03125	.30946	-.571	29	.572	0	1.000
Positive vs. Neutral	-.03125	.17678	-1.000	29	.325	-1	.317
**2-back**							
Negative vs. Positive	.03125	.82244	.215	29	.831	0	1.000
Negative vs. Neutral	.31250	.85901	2.058	29	**.048**	-1.705	.088
Positive vs. Neutral	.28125	111.397	1.428	29	.163	-1.429	.153
**3-back**							
Negative vs. Positive	-.18750	102.980	-1.030	29	.311	-1.035	.301
Negative vs. Neutral	-.46875	.71772	-3.695	29	**.001**	-2.977	**.003**
Positive vs. Neutral	.28125	.85135	1.869	29	.071	-1.642	.101

### 3. FNIRS Results

#### 3.1 Oxygenated Haemoglobin

Four test subjects had to be excluded due to technical artefacts, so all analyses were based on 26 test subjects.

**Figure 3:**
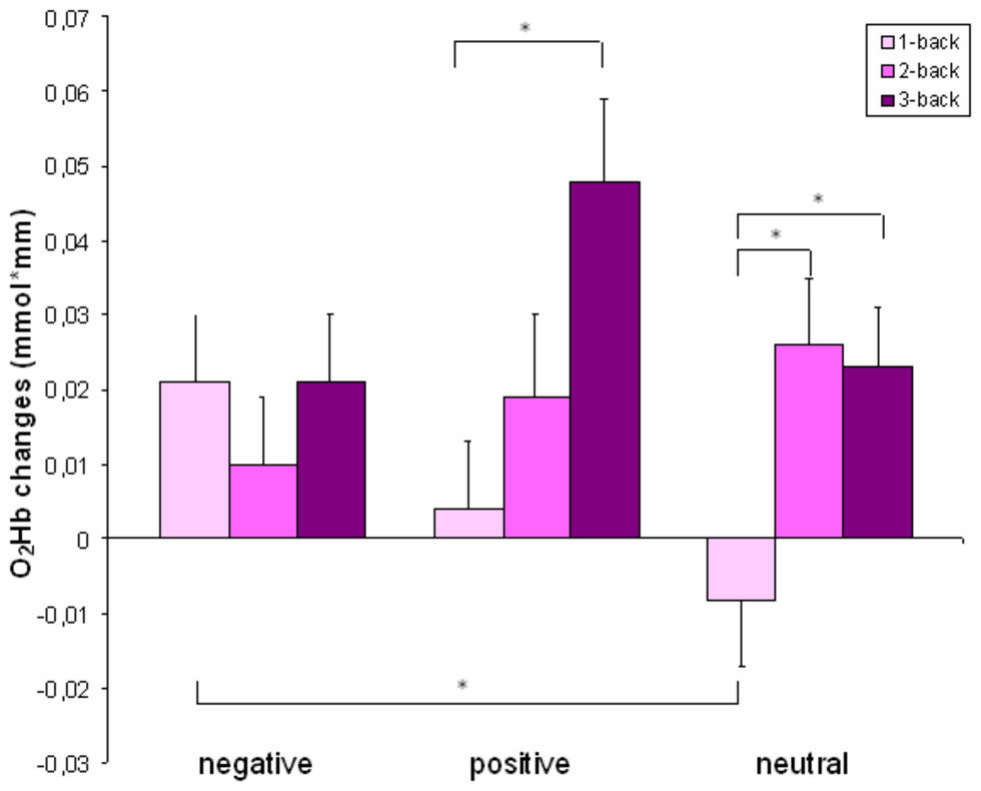
O_2_Hb changes. Changes in the oxygenated blood concentration in the ROI. Bars indicate the standard error. * indicates significant differences between difficulty levels (p<0.05).

The 3x3 ANOVA for the oxygenated haemoglobin revealed a significant main effect for the difficulty level (F(2, 50) = 4.2, *p* = .021), no main effect for emotional valence (F(2, 50) = 1.2, *p* = .3), but a significant interaction effect (F(4, 100) = 2.6, *p* = .039) indicating that the word valence influences the oxygenation in a given difficulty level differentially (Figure 3 and Table 4). A repeated measures ANOVA with the additional factor hemisphere revealed no differences in activation between the two hemispheres (F < 1).

**Table 4:** Post hoc t-tests for the ROI (consistent of BA9, BA10, BA46) in the oxygenated condition.

**Oxy Data**						
**Negative**	**mean**	**SD**	**SE**	**T**	**df**	**Sig. (2-sided)**
1-back vs. 2-back	.01060	.06476	.01270	.835	25	.412
1-back vs. 3-back	-.00068	.07008	.01374	-.050	25	.961
2-back vs. 3-back	-.01128	.04665	.00915	-1.233	25	.229
**Positive**						
1-back vs. 2-back	-.01527	.06554	.01285	-1.188	25	.246
1-back vs. 3-back	-.04409	.07920	.01553	-2.838	25	**.009**
2-back vs. 3-back	-.02882	.08105	.01590	-1.813	25	.082
**Neutral**						
1-back vs. 2-back	-.03364	.07201	.01412	-2.382	25	**.025**
1-back vs. 3-back	-.03067	.06784	.01331	-2.305	25	**.030**
2-back vs. 3-back	.00297	.06343	.01244	.239	25	.813
**1-back**						
Negative vs. Positive	.01684	.06863	.01346	1.251	25	.222
Negative vs. Neutral	.02806	.05280	.01036	2.710	25	**.012**
Positive vs. Neutral	.01122	.06448	.01265	.887	25	.383
**2-back**						
Negative vs. Positive	-.00903	.05432	.01065	-.847	25	.405
Negative vs. Neutral	-.01618	.04352	.00854	-1.895	25	.070
Positive vs. Neutral	-.00715	.06501	.01275	-.561	25	.580
**3-back**						
Negative vs. Positive	-.02656	.07244	.01421	-1.870	25	.073
Negative vs. Neutral	-.00192	.05900	.01157	-.166	25	.869
Positive vs. Neutral	.02464	.06524	.01279	1.926	25	.066

Significant channels are highlighted in bold.

#### 3.2 Deoxygenated Haemoglobin

**Figure 4:**
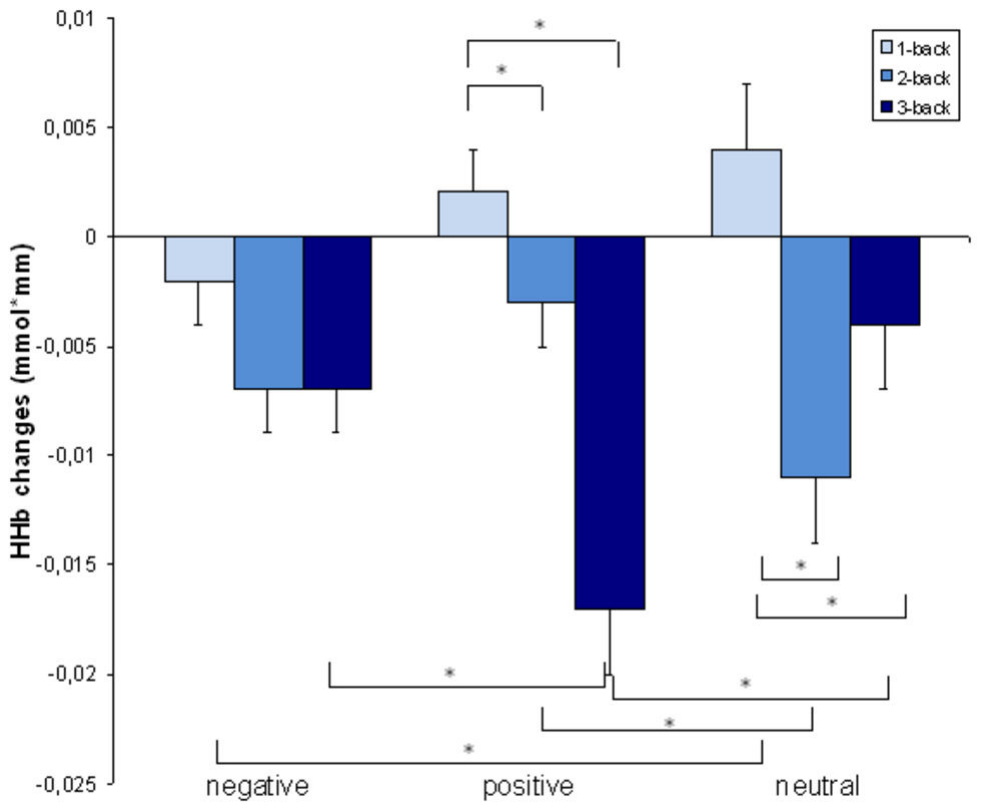
HHb changes. Changes in the deoxygenated blood concentration in the region of interest. Bars indicate the standard error. An asterisk indicates significant differences between conditions.

The 3x3 ANOVA for the deoxygenated haemoglobin replicated the pattern described above. Again, there was a significant main effect for the difficulty level (F(2, 50) = 14.5, *p* < .001), no significant main effect for the emotional valence (F < 1) and a significant interaction effect (F(4, 100) =7.3, *p* < .001) (Figure 4 and Table 5). Again, the repeated measures ANOVA for the right and left hemisphere revealed no differences in activation between the two hemispheres (F < 1).

**Table 5:** Post hoc t-tests for the ROI in the deoxygenated condition.

**Deoxy Data**						
**Negative**	**mean**	**SD**	**SE**	**T**	**df**	**Sig. (2-tailed)**
1-back vs. 2-back	.00460	.01420	.00278	1.652	25	.111
1-back vs. 3-back	.00451	.01471	.00289	1.563	25	.131
2-back vs. 3-back	-.00009	.01223	.00240	-.037	25	.971
**Positive**						
1-back vs. 2-back	.00512	.01350	.00265	1.934	25	.065
1-back vs. 3-back	.01838	.01987	.00390	4.716	25	**.000**
2-back vs. 3-back	.01326	.01804	.00354	3.749	25	**.001**
**Neutral**						
1-back vs. 2-back	.01545	.01701	.00334	4.632	25	**.000**
1-back vs. 3-back	.00839	.01673	.00328	2.558	25	**.017**
2-back vs. 3-back	-.00706	.01836	.00360	-1.960	25	.061
**1-back**						
Negative vs. Positive	-.00390	.01627	.00319	-1.223	25	.233
Negative vs. Neutral	-.00630	.01562	.00306	-2.055	25	**.050**
Positive vs. Neutral	-.00239	.01632	.00320	-.748	25	.462
**2-back**						
Negative vs. Positive	-.00338	.01228	.00241	-1.403	25	.173
Negative vs. Neutral	.00456	.01334	.00262	1.742	25	.094
Positive vs. Neutral	.00794	.01585	.00311	2.553	25	**.017**
**3-back**						
Negative vs. Positive	.00997	.01937	.00380	2.625	25	**.015**
Negative vs. Neutral	-.00241	.01703	.00334	-.722	25	.477
Positive vs. Neutral	-.01238	.01423	.00279	-4.436	25	**.000**

Significant channels are highlighted in bold.

### 4. EEG Results

**Figure 5:**
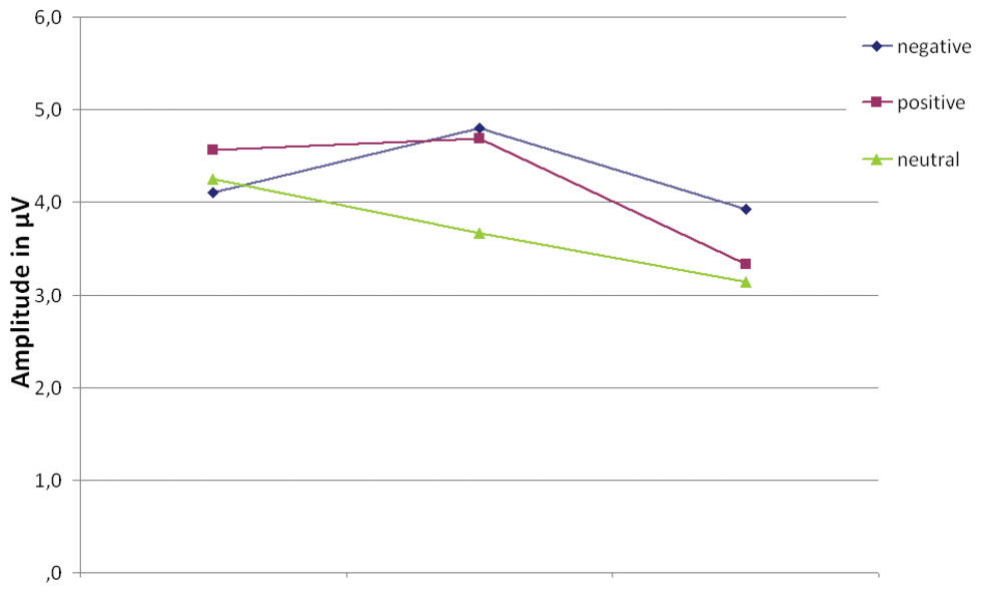
LPP Amplitude. Mean Amplitude of the early LPP for the different valences (positive, negative and neutral) in the different task difficulties. An asterisk indicates significant differences in the amplitude for the different valences.

**Figure 6:**
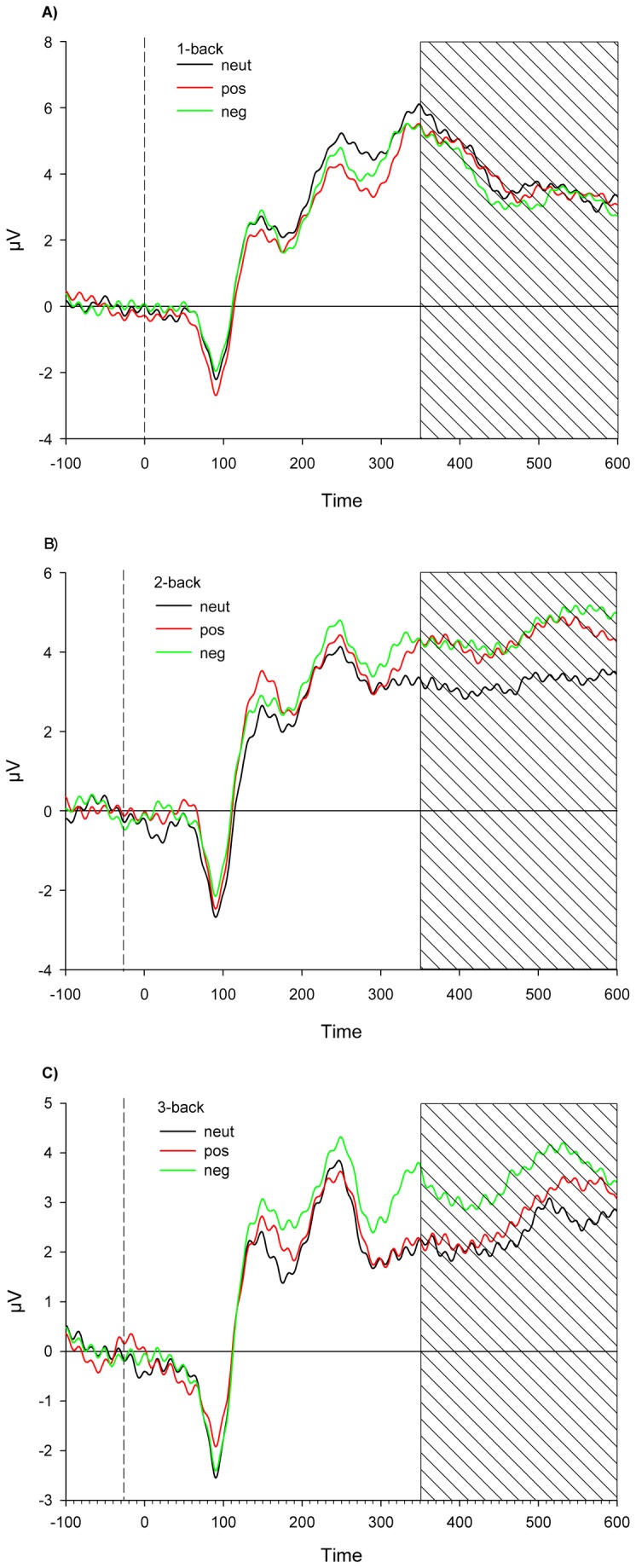
ERP curves. Stimulus locked ERPs averaged at Cz, Pz, CPz, CP1 and CP2 for the valences neutral, negative and positive for the 1-back condition (a), the 2-back condition (b) and the 3-back condition (c).

The 3x3 ANOVA revealed a significant main effect for the difficulty level (F(2, 58) = 6.9, *p* = 0.002), a significant main effect for the emotional valence (F(2, 58) =5.4, *p* = .007) and a significant interaction effect (F(4, 116) = 2.7, *p* = .036). This indicates that the difficulty level of the working memory task modulates the response to emotionally salient words. Results from the post hoc t-tests can be seen in Table 6 and are also illustrated in Figures 5 and 6 A, B and C, and show that in the 1-back task, the LPP is not different between the emotional valence of the words, whereas in the 2-back task, the LPP differentiates between neutral and emotional words but not between the emotional valences. Finally, in the 3-back task it is only possible to dissociate the negative valence within the LPP.

**Table 6:** Post-hoc t-tests for the LPP in the different conditions.

**EEG Data**						
**1-back**	**mean**	**SD**	**SE**	**T**	**df**	**Sig. (2-taled)**
Negative vs. Positive	0.4594067	2.0654595	0.3770996	1.218	29	.233
Negative vs. Neutral	-0.14945	1.5618909	0.285161	-0.52	29	.604
Positive vs. Neutral	0.3099567	2.0309451	0.3707981	0.836	29	.41
**2-back**						
Negative vs. Positive	-0.1111	1.6167969	0.2951854	-0.38	29	.709
Negative vs. Neutral	1.13178	1.7376721	0.3172541	3.567	29	**.001**
Positive vs. Neutral	1.02068	2.2039156	0.4023781	2.537	29	**.017**
**3-back**						
Negative vs. Positive	-0.5886233	2.0386779	0.37221	-1.58	29	.125
Negative vs. Neutral	0.77812	1.6077277	0.2935296	2.651	29	**.013**
Positive vs. Neutral	0.1894967	1.6152359	0.2949004	0.643	29	.526
**negative**						
1-back vs. 2-back	-.1215833	2.1107219	.3853633	-.316	29	.755
1-back vs. 3-back	1.2296567	2.7527622	.5025833	2.447	29	**.021**
2-back vs. 3-back	1.3512400	2.2330758	.4077020	3.314	29	**.002**
**Positive**						
1-back vs. 2-back	-.6920900	2.0933981	.3822005	-1.811	29	.081
1-back vs. 3-back	.1816267	1.8441168	.3366881	.539	29	.594
2-back vs. 3-back	.8737167	1.6660780	.3041828	2.872	29	**.008**
**Neutral**						
1-back vs. 2-back	.5891400	2.1311338	.3890900	1.514	29	.141
1-back vs. 3-back	1.1091967	2.1974087	.4011901	2.765	29	**.010**
2-back vs. 3-back	.5200567	1.9211196	.3507469	1.483	29	.149

Significant differences are highlighted in bold.

## Discussion

The first aim of this study was to investigate whether valence of words carefully matched for arousal has an effect on working memory performance. Error data revealed a modulation of performance by word valence. More errors were produced when the word had a negative valence, and that effect was most pronounced in the most difficult 3-back task. Reaction time did not resemble error data, as it increased only as a function of the increasing working memory load. It has been shown that reaction time in an emotional Stroop test was mainly influenced by arousal and not valence [9]. The words used in this study were selected based on their arousal similarities. This could be an explanation for the lack of reaction time effects. However, this would only apply to differences between the emotional conditions, but not to differences with neutral stimuli. Another explanation for this finding could be the small trial size, which may prevent finding significance when effects are small.

The changes in the blood oxygenation level revealed a main effect of task difficulty and an interaction effect for difficulty by emotional valence. Even though the emotional word itself does not change the blood oxygenation as a mean of the valence, it seems to influence the brain activation nonetheless in such a way that the higher the load, the more pronounced the differences between the emotional valences. In detail, while the positive valence does not seem to have any effect on the oxygenated blood level increases, and the brain activation seems to be the comparable to a regular n-back task [32,33], the negative words seem to influence the activation levels so that no change between the different load conditions is detectable. The same pattern becomes even more pronounced in the deoxygenated blood flow changes. This effect of reduced blood flow changes in reaction to negatively valenced stimuli has also been shown by other groups [40] with emotional pictures, who found reduced activation in the dlPFC for the negative pictures. However, the neutral words induce an unexpected pattern of activation changes, both in oxygenated and deoxygenated haemoglobin levels. From the 1-back to the 2-back condition they increase activation in a manner similar to the positive words. However, as the task becomes even more complicated, the neutral words seize to increase activation: in the 3-back task they cannot be differentiated from the negative words anymore. We expected the neutral words to elicit activation patterns that show an increase in activation from 1-back to 2-back to 3-back, as can be seen in working memory tasks using letters [32,33]. We have no explanation for the discrepant findings so far. We tested whether some participants might have had different evaluation criteria for the words, and aimed to identify outliers within the rating of the words, but the visual inspection could not account for the findings. It is also possible that the different arousal for the neutral words is causing part of the unexpected pattern, and together with the very low difficulty level of the task itself could provide an explanation.

We next analyzed the LPP, and found significant effects of emotion, but also significant effects of difficulty or working memory load and a significant interaction between both. In the 2-back task, the LPP distinguishes between neutral and emotional words very clearly, whereas in the 1-back task that distinction is not detected. In the 3-back task, the only distinction visible in the LPP is for the negative words. It seems that in the easier 2-back condition, the arousal of the word is important for the distinction the LPP can provide, whereas in the most difficult 3-back condition, valence is the key to distinction. We can therefore show that the LPP as well can be influenced by the difficulty level in a working memory task, and differentially for the different emotional valences [14]. However, contrary to earlier findings by MacNamara and colleagues [14], when using the stimulus itself as the carrier of the emotional valence, the LPP first becomes larger with increasing working memory load, and only when the load increases further, we observed a decrease in the LPP. That may be due to the different paradigms used, as the study by MacNamara and colleagues used emotional pictures, and they were only used as distractors, not as the stimuli to be remembered themselves, which were strings of letters. But nonetheless, the main finding of the LPP results in our study as well as in that of MacNamara et al. [14] is that emotion processing is influenced by a simultaneously occurring working memory task, which takes away more and more capacity from emotion processing as the load increases.

Our study adds to the still comparatively small amount of literature on emotion processing measured with fNIRS [41-44]. Interestingly, this is not the first finding of early facilitation by emotional content. For example, Plichta et al. [44] found changes in activation of the auditory cortex depending on whether or not participants heard a pleasant, unpleasant or neutral voice. These results seem to suggest fNIRS as an easily applicable, reliable method to investigate the influence of emotion on other executive functions.

Taken together, our data suggest that it is not only arousal that plays a role in the modulation of working memory but also the valence of the emotional content stored in working memory may play a role in its function. This underlines and extends findings from Levens and Phelps [12] who used a similar paradigm to show emotion effects on working memory, and furthermore points to the importance of the valence itself.

By integrating the two methods, we conclude that the emotional content of words is influencing working memory performance measured by prefrontal blood flow changes, and that working memory performance is influencing the processing of emotional stimuli. If working memory and the processing of emotional stimuli influence each other, this has implications for clinical research centering around affect disorders, where both working memory and emotion regulation processes are disturbed. Future research should address the question of the molecular basis of these prefrontal functions.

### Limitations

First, the number of trials was rather small. There is a certain trade-off between the time the fNIRS probeset can be worn without being uncomfortable and the time needed to collect enough trials for both fNIRS and EEG measurements. Thus, the trial number could have been too small to detect stable effects. However, since the a priori hypotheses could be largely supported, we are confident that this compromise can be considered acceptable.

Second, we used only few electrode sites to record the LPP. This was a result of the simultaneous fNIRS and EEG recordings. As the fNIRS probeset and the fibers covered most of the forehead, we could not measure EEG from these sides. However, since it is known that the LPP amplitude is largest over parietal sites, we reckon the recording from these electrode sites to be rather valid. The use of combined fNIRS-EEG caps with holders for optodes and electrodes in future studies will help to estimate the influence of the setting.

Third, another limitation may be the EEG recording device. The Quickamp (Brainproducts, Munich, Germany) device references to a common average reference, which cannot be changed. We tried to overcome this by re-referencing offline after data collection.
